# Experimental Burn Induction in Laboratory Animals: A Scoping Review of Methods, Reproducibility, Operator-Dependent Variability, and Relevance to Soft Tissue Reconstruction and Repair

**DOI:** 10.3390/bioengineering13060601

**Published:** 2026-05-22

**Authors:** Antonios Kyriakopoulos, Michalis Katsimpoulas, Vasilios Kyriakopoulos, Evangelos Felekouras, Stratigoula Sakellariou, Ioannis Kouris, Alexandros Charalabopoulos

**Affiliations:** 1 Plastic Surgery Department, Evaggelismos General Hospital of Athens, 10676 Athens, Greece; 2 Experimental Surgery, Regenerative Medicine Preclinical Research, Experimental Surgical Unit, Center of Clinical, Experimental Surgery and Translational Research, Biomedical Research Foundation of the Academy of Athens, 11527 Athens, Greece; mkatsiboulas@bioacademy.gr; 3 Physiotherapy Department, Athens Technological Educational Institute, University of West Attica, 12243 Athens, Greece; 4 1st Surgical Department, Laiko Hospital, Medical School, National and Kapodistrian University of Athens, 11527 Athens, Greeceacharalabopoulos@yahoo.com (A.C.); 5 1st Department of Pathology, Medical School, Laiko Hospital, National and Kapodistrian University of Athens, 11527 Athens, Greece; 6 Iodine Therapy Department, General Oncological Hospital of Kifisia “Oi Agioi Anargyroi”, 14564 Athens, Greece

**Keywords:** burn injury, animal models, experimental burns, reproducibility, repeatability, operator-dependent variability, burn depth, scoping review, wound healing, thermal injury

## Abstract

Background: Experimental animal models remain central to burn research and soft-tissue reconstruction/repair, but method heterogeneity compromises reproducibility, comparability, and translation for depth/area endpoints. Objective: We aimed to map burn-induction methods and examine reproducibility, intentional depth modulation, wound-area stability, validation, and operator-dependent variability. Methods: A PRISMA-ScR review, informed by JBI guidance, was conducted without registration but with predefined questions, criteria, and charting domains. PubMed/MEDLINE, Scopus, Web of Science, Embase, and Google Scholar were searched from inception to 30 January 2026. Eligible studies were English peer-reviewed full-text original in vivo animal studies. Two reviewers independently screened records; one charted data, another checked it. Evidence was mapped by modality, exposure-control architecture, validation, and operator-sensitive steps. Results: Studies varied by species, modality, device design, exposure settings, and severity verification. Modalities were contact, scald, steam, and radiant/infrared. Wound area was more reproducible than depth, which depended on temperature, duration, force/pressure, geometry, equilibration, anatomical site, and assessment timing. Histopathology was the main standard, sometimes complemented by morphometry, optical, or perfusion techniques. Operator-sensitive variability involved force, alignment, contact stability, template integrity, exposure geometry, source stability/environmental control. Conclusions: Burn induction is a measurement-system problem; constraining operator-sensitive variables, predefined validation timing, and quantitative variability reporting may improve validity, comparability, and translation.

## 1. Introduction

Burn injury remains a major global health problem, associated with substantial mortality, long-term disability, prolonged rehabilitation, and considerable healthcare costs. The World Health Organization estimates that approximately 180,000 deaths occur annually from burns, with the greatest burden in low- and middle-income countries [1,2]. The impact of burns, however, extends well beyond mortality. Non-fatal injuries may lead to hypertrophic scarring, contractures, chronic pain, infection, functional limitation, and psychosocial morbidity, highlighting the need for accurate assessment of burn severity and for preclinical models that are relevant to clinical care [3]. Beyond their role in elucidating burn pathophysiology, experimental burn models are increasingly relevant to studies of wound closure, skin replacement, tissue regeneration, and scar modulation, thereby linking burn research directly to soft tissue reconstruction and repair [4,5].

In clinical burn care, two variables are central to early management and subsequent outcome: total body surface area (TBSA) and burn depth. These parameters guide resuscitation, influence infection risk, inform the need for excision and grafting, and are closely linked to re-epithelialization time and scar quality [6,7]. Burn depth has historically been assessed by visual inspection and tactile examination. Although these methods remain embedded in routine practice, their diagnostic performance is variable and strongly dependent on clinical experience. By contrast, objective technologies have demonstrated substantially greater accuracy in burn-depth assessment. In a meta-analysis of laser Doppler imaging (LDI), pooled sensitivity and specificity for burn-depth diagnosis were reported as 91% and 96%, respectively, with an area under the receiver operating characteristic curve of 0.98, supporting the value of objective perfusion-based assessment in severity classification and treatment planning [8]. Serial analyses have also shown that the accuracy of both clinical examination and imaging varies with time after injury, which emphasizes that burn depth is not a static attribute measured only at the moment of thermal exposure [9]. This clinical reality has direct translational implications. Preclinical burn models should therefore generate injuries with quantifiable and reproducible severity, and should validate injury depth at clearly defined post-burn timepoints. This consideration is particularly important when such models are used to evaluate reconstructive or regenerative interventions, because wound depth and healing trajectory are central determinants of grafting strategy, dermal replacement, and scar-related outcomes [10].

Animal models remain indispensable in burn research because they allow controlled investigation of mechanisms that are difficult to isolate in clinical populations, including inflammatory signaling, microvascular dysfunction, bacterial colonization, hypermetabolism, and tissue repair [11,12]. In this setting, animal burn models serve not only as systems for studying injury mechanisms, but also as preclinical test platforms for skin substitutes, dermal matrices, and cell-based therapies used in soft tissue reconstruction and repair [13]. An animal model’s translational value relies on both species selection and injury production. Choosing the correct species affects construct validity and external validity. In burn research, porcine models are often preferred because porcine skin shares several important features with human skin, including comparable thickness, dermal architecture, adnexal structures, and aspects of healing behavior. Rodent models remain widely used because they are less expensive, experimentally scalable, and compatible with transgenic and immunologic approaches. This is particularly relevant for translational reconstruction research, since porcine skin more closely approximates human skin architecture and wound-healing behavior, making it especially valuable for studies of grafting, dermal substitutes, and regenerative skin repair [14].

At the same time, ethical justification and methodological rigor must be considered together. Contemporary in vivo research is expected to align with the principles of Replacement, Reduction, and Refinement, while also meeting accepted standards for transparent reporting and reproducible experimental design [15]. The ARRIVE 2.0 guidelines explicitly emphasize rigorous reporting of animal characteristics, procedural details, outcome assessment, and sources of bias, all of which are directly relevant to burn-model standardization [16,17].

A major barrier to cross-study comparison in the burn-model literature is methodological heterogeneity in injury induction. Protocols that appear similar at the level of nominal temperature and exposure duration may nevertheless produce substantially different injuries because thermal dose at the tissue interface is shaped by multiple additional variables. These include contact-surface temperature, duration of exposure, applied force or pressure, contact geometry, thermal equilibration of the device, distance from the heat source in non-contact systems, local skin thickness, perfusion, hydration, and anatomic site. As a result, reproducibility in experimental burn induction should be approached as a measurement-system problem rather than as a simple temperature–time prescription.

This distinction is clarified by established metrological terminology. Repeatability refers to the closeness of results obtained under the same operating conditions, typically when the same operator uses the same instrument and protocol in the same setting. Reproducibility refers to the closeness of results obtained when relevant conditions vary, such as operator, laboratory, or equipment configuration. Applied to burn induction, these concepts provide a useful framework for understanding why nominally comparable protocols can yield different wound depths and areas across studies or between operators [18,19]. In this context, operator-dependent variability is not a minor procedural nuisance. It is a central determinant of internal validity. Any manual component that affects pressure, alignment, timing, contact stability, or thermal conditioning may alter energy transfer and thereby modify injury severity.

Several device designs have been developed to constrain exposure variables more tightly. These include contact applicators with fixed geometry, systems using calibrated mass or force control, circulating-water scald platforms, rigid template-based exposure windows, and steam or radiant systems with standardized delivery geometry. Certain designs, in a few cases, have been improved and provide a greater level of control over the size and/or depth of burn injury [20]. In rodent contact systems, graded combinations of temperature and exposure time have produced distinct histological depths of injury, supporting the idea that burn severity can be tuned when the exposure system is adequately controlled [21,22]. Similar observations have been reported in porcine studies, where engineering refinements improved thermal stability and between-replicate consistency, while also drawing attention to the importance of anatomical site and post-burn timing when depth outcomes are interpreted [23,24]. Taken together, these studies suggest that standardization cannot be assumed from device description alone. It must be demonstrated through objective validation of the injury produced and through explicit reporting of variability.

Previous reviews have summarized experimental burn models, porcine burn conditions, clinical burn-depth assessment, wound repair, skin replacement, and regenerative strategies after burn injury. These works are valuable, but they are generally organized around pathophysiology, treatment platforms, species selection, or clinical translation. By contrast, the present review focuses specifically on the burn-induction step itself as a potential source of experimental measurement error. Its originality lies in mapping how heat-transfer modality, exposure-control architecture, operator-sensitive procedural variables, anatomical substrate, validation timing, and quantitative reporting of variability influence the reproducibility of burn depth and wound area. The relevance to soft tissue reconstruction and repair is therefore methodological and translational rather than based on a systematic comparison of reconstructive interventions: inconsistent initial burn depth can plausibly confound downstream assessment of graft take, dermal substitute integration, contraction, scar formation, and regenerative healing.

Against this background, a structured mapping of the literature is needed. The critical methodological question is not simply which animal burn models exist, but which induction methods permit reproducible control of burn depth and surface area, which variables are operator-sensitive, and how consistently injury severity is validated across studies. The purpose of this review is therefore to examine experimental burn-induction methods in laboratory animals with emphasis on four domains: reproducibility, intentional modulation of burn depth, stability of wound area, and operator-dependent procedural sources of variability. By synthesizing this evidence, the review aims to provide a practical framework for selecting and reporting burn-induction methods and to improve interpretation and comparability across preclinical studies.

## 2. Materials and Methods

### 2.1. Review Design and Reporting Framework

This study was conducted as a scoping review with structured evidence mapping. This design was selected because the literature on experimental burn induction in laboratory animals is methodologically heterogeneous and not readily amenable to effect-based quantitative synthesis. Important variation exists across animal species, burn-induction modalities, device architecture, exposure parameters, methods used to define injury severity, and the timepoints selected for outcome verification. Under these conditions, the primary objective is to map how the field has approached burn induction and reproducibility rather than to derive a pooled estimate from non-equivalent experimental systems [25,26,27]. The review was reported in accordance with the PRISMA Extension for Scoping Reviews (PRISMA-ScR) and was informed by current Joanna Briggs Institute guidance for scoping reviews [25,26]. These frameworks were used to structure the review questions, eligibility criteria, literature search, source selection, data charting, and synthesis of findings.

No formal review protocol was registered or published before commencement of the review. However, the review was guided by an a priori methodological framework defined before study selection. This framework specified the review questions, the Population–Concept–Context eligibility structure, the inclusion and exclusion criteria, the information sources, and the core data-charting domains. These predetermined domains included animal and model characteristics, induction modality, device architecture, exposure parameters, validation method, validation timing, reproducibility metrics, and operator-sensitive procedural variables. During full-text review and data charting, minor refinements were made at the level of extraction granularity in order to capture modality-specific procedural details, such as force or pressure in contact models, template seating in scald models, and source-to-skin geometry in steam or radiant models. These refinements did not alter the review questions, eligibility criteria, population, concept, context, language restrictions, publication-type restrictions, or the primary methodological focus of the review. They were made to improve the completeness and consistency of evidence mapping across heterogeneous experimental systems.

### 2.2. Review Questions

The review was designed to address four related methodological questions. First, which experimental methods have been used to induce cutaneous burns in laboratory animals? Second, which controllable exposure parameters are reported as determinants of burn depth and burn surface area? Third, what evidence is available regarding repeatability, reproducibility, and operator-dependent variability across these methods? Finally, what methods have been used to validate the severity of an injury, and at what time after the burn occurred were these assessments made? These questions were selected to keep focused on model development and methodological performance. The purpose of this review was not to compare how post-burn interventions have been completed, but to investigate the generation, standardization, and determination of the thermal injury itself in the preclinical literature.

### 2.3. Eligibility Criteria

Eligibility criteria were established a priori using a Population–Concept–Context framework appropriate for scoping-review methodology [26]. The population of interest comprised in vivo laboratory animals of any species in which a cutaneous burn was experimentally induced under controlled conditions. Studies using rodents, pigs, rabbits, or other laboratory species were eligible if the model involved deliberate creation of a skin burn and the method was described with sufficient procedural detail. For the purposes of eligibility, “sufficient procedural detail” was defined as reporting, at minimum, the animal species, the anatomical site or target region, the burn-induction modality, the heat source or device type, the target temperature or source setting, the exposure duration, the intended wound size or exposure area when applicable, and at least one method used to confirm burn creation or injury severity. Studies were excluded when the burn was described only as a generic thermal injury without enough information to determine how the injury had been produced. Studies that met the minimum induction-detail threshold but lacked information on secondary reproducibility variables, such as operator training, interface temperature, or calibration procedures, were retained and charted as incompletely reported for those domains. The concept of interest was the experimental induction of cutaneous thermal injury. Eligibility criteria for studies included descriptions of multiple modes of induction, including contact burns, scalds, steam, radiation, and infrared burns (and/or associated thermal induction methods) and sufficient methodological information to evaluate the external validity of their exposure system. The studies of particular interest were studies that provided information about the controllable variables in the induction process, the target area of the wound produced, and the manner in which the injury had been validated.

The review included studies conducted in preclinical and translational laboratory settings, with no restriction on country of origin. Only full-text, peer-reviewed original articles published in English were considered eligible. Conference abstracts were excluded because they generally did not provide enough procedural detail to support reliable methodological charting. Review articles were not included in the extraction dataset, although their reference lists were examined to identify additional primary studies that met the eligibility criteria. Studies were excluded if they did not involve an in vivo animal model, relied exclusively on human clinical material, focused on burn pathophysiology or treatment without describing the induction method, examined non-cutaneous thermal injury, or lacked sufficient detail to determine how the burn had been created.

### 2.4. Information Sources and Search Strategy

A structured literature search was developed to maximize retrieval of animal studies describing experimental burn induction while maintaining specificity for reproducible preclinical protocols. Electronic searches were performed in PubMed/MEDLINE (National Library of Medicine, Bethesda, MD, USA), Scopus (Elsevier, Amsterdam, The Netherlands), Web of Science Core Collection (Clarivate, London, UK), Embase (Elsevier, Amsterdam, The Netherlands), and Google Scholar (Google LLC, Mountain View, CA, USA) from database inception to 30 January 2026. The search strategy combined controlled vocabulary, where available, with free-text terms related to burn injury, thermal injury, animal models, and experimental induction. Modality-specific terms were also incorporated in order to capture studies using contact, scald, steam, radiant, and infrared methods. Search terms included combinations of burn, thermal injury, animal model, experimental induction, contact burn, scald burn, steam burn, radiant burn, infrared burn, device, method, and protocol. The search syntax was adapted to the indexing structure and retrieval logic of each database.

To improve completeness, the reference lists of all included studies were examined manually. Citation chasing was also performed through relevant review articles identified during screening, although those reviews were not used as primary evidence sources for data charting.

### 2.5. Selection Process

All records identified through the database search were imported into Zotero (version 9.0.3, 64-bit; Corporation for Digital Scholarship, Falls Church, VA, USA) for reference management and deduplication. Duplicate records were removed electronically and then checked manually to reduce the risk of excluding citations with incomplete or inconsistently formatted metadata. Study selection was carried out in two stages. Titles and abstracts were screened first, and all potentially eligible articles then underwent full-text review. Both stages were completed independently by two reviewers. Any disagreements were resolved through discussion until consensus was reached. This approach was used to improve consistency in study selection and to reduce the chance that relevant articles would be excluded on the basis of a single reviewer’s judgment.

### 2.6. Data Charting Process

Data charting was carried out using a structured extraction framework developed specifically for this review. The framework was intended to capture the methodological features most likely to influence heat transfer, injury severity, and comparability across studies. The focus was therefore not only descriptive, but also analytical. The first reviewer completed data extraction and a second reviewer checked the extraction in its entirety. Discrepancies were settled through a review of the original article and discussion with respect to arriving at an agreement about how to proceed. Where a study reported more than one induction condition, such as different temperature–time combinations, alternative device configurations, or multiple validation timepoints, these data were charted separately when needed in order to preserve meaningful methodological distinctions.

This approach was considered especially important because reproducibility claims were often linked not to the device in general, but to particular exposure settings or to specific post-burn assessment intervals.

### 2.7. Data Items

For clarity and transparency, the variables extracted from each study are presented in Table 1.

### 2.8. Operational Definitions

Because the central objective of the review was methodological mapping, operational definitions were specified in advance in order to standardize charting and interpretation across a heterogeneous evidence base. Repeatability was defined as the closeness of repeated results obtained under the same conditions, typically when the same operator used the same device and protocol in the same setting. Reproducibility was defined as the closeness of results obtained when one or more relevant conditions differed, such as operator, experimental session, or device setup. These terms were used in a practical manner consistent with accepted measurement terminology.

Operator-dependent variability was defined as variability plausibly attributable to manual procedural steps capable of influencing heat transfer or exposure geometry. Such steps included timing of application, force or pressure, alignment, contact stability, template seating, immersion handling, nozzle positioning, and the interval between device heating and tissue contact. Depth tunability referred to evidence that injury severity could be intentionally modulated by altering controllable exposure variables such as temperature, time, force, or distance. Area stability referred to the consistency with which the intended wound area was reproduced across repeated applications.

These definitions were used because nominally similar burn protocols frequently differed in practice at exactly these points. Accordingly, the charting process focused not only on the stated design of each method, but also on which variables were actually constrained, measured, or left to operator control.

### 2.9. Framework for Interpreting Methodological Control and Strength of Evidence

Because this was a scoping review rather than an intervention-effect review, no formal risk-of-bias tool was applied. However, to avoid unstructured qualitative appraisal, methodological features were interpreted using a predefined descriptive framework focused on exposure control and evidence type. For each modality, the extracted evidence was categorized according to: (i) whether key exposure variables were mechanically or procedurally constrained; (ii) whether injury severity was validated objectively; (iii) whether variability was reported quantitatively; and (iv) whether operator-dependent effects were directly measured or inferred from operator-sensitive procedural steps. Evidence was described as directly measured when a study experimentally tested a variable such as force, duration, temperature, distance, or observer agreement and reported a corresponding outcome. Evidence was described as quantitatively reported when dispersion or reliability metrics such as SD, CV, ICC, kappa, or range were provided. Evidence was described as inferred when a procedural variable was plausibly operator-sensitive but was not directly tested as an independent factor. This framework was used for descriptive mapping only and was not intended to grade certainty of evidence or rank studies by risk of bias.

### 2.10. Data Synthesis

A formal meta-analysis was not performed. This decision was made a priori and reflected the substantial heterogeneity of the included literature. The studies differed in species, tissue characteristics, induction modality, device architecture, thermal interface conditions, definitions of burn severity, post-burn validation timepoints, and the statistical measures used to report variability. Under such circumstances, quantitative pooling would have combined outcomes that were not sufficiently equivalent in either biological meaning or methodological construction. In this setting, pooled estimates would have had limited interpretability and could have obscured, rather than clarified, the main methodological issues [26].

The evidence was therefore synthesized through structured narrative analysis combined with evidence mapping. Studies were grouped first by induction modality and then, where possible, by the degree to which key operator-mediated variables appeared to be constrained by the device or protocol. Within these groups, the synthesis focused on controllable exposure parameters, evidence for intentional modulation of burn depth, methods used to validate injury severity, reported indicators of repeatability or reproducibility, and procedural steps vulnerable to operator influence.

Reported reproducibility metrics, including standard deviations, coefficients of variation, kappa statistics, and intraclass correlation coefficients, were tabulated descriptively and interpreted in relation to device design, exposure control, and timing of verification. No attempt was made to pool these measures across studies because they were derived from non-uniform outcome definitions and non-comparable assessment schedules. A formal risk-of-bias assessment was not undertaken. This was consistent with the purpose of the review, which was to map methodological approaches and characterize how reproducibility had been conceptualized and reported, rather than to estimate pooled intervention effects or compare therapeutic efficacy. Within PRISMA-ScR, critical appraisal is optional and should be undertaken when it is directly relevant to the review objective. In the present review, methodological limitations were nevertheless recorded during charting when they were directly relevant to interpretation, particularly when key variables such as interface temperature, force, anatomical site, operator training, or validation timing were incompletely reported.

## 3. Results

### 3.1. Overview of Included Evidence

The studies included in this review were methodologically varied. Both small- and large-animal models were represented, but rats and mice were used most often, particularly in studies focused on mechanistic questions or on scalable preclinical testing. Porcine models appeared less frequently, but were more common in studies with a clearer translational emphasis. Across the literature, the main burn-induction approaches were contact burns, scald burns, steam-based systems, and radiant or infrared models [28,29,30,31,32,33,34,35,36,37,38,39,40].

Even so, several shared methodological aims were apparent across studies. A central objective was the generation of a predefined depth of injury. Many studies also sought to produce a wound area that was both stable and reproducible. Another recurring aim was to reduce uncontrolled variation related to force, timing, positioning, or thermal instability during exposure. Finally, a substantial part of the literature focused on how injury severity was verified. Histopathology was the most commonly used method, although some studies also incorporated quantitative morphometry, planimetry, perfusion-sensitive imaging, or non-contact optical tools to strengthen objective assessment [34,35,40,41].

**Figure 1:**
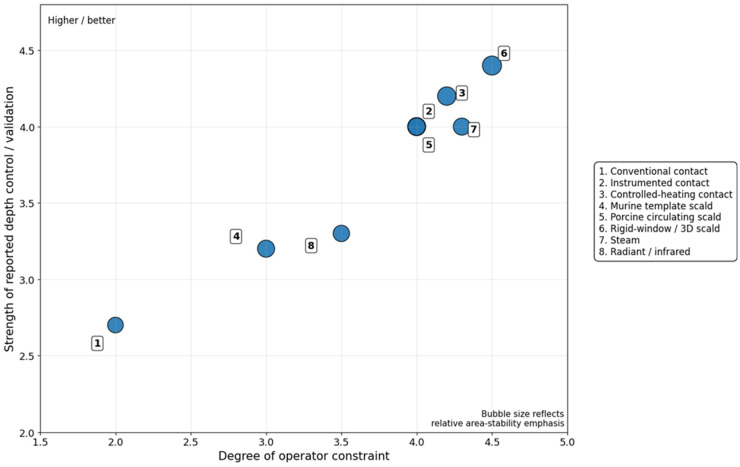
Qualitative evidence map by induction modality.

Viewed as a whole, the evidence indicated that experimental burn severity was not determined by temperature and exposure time alone. Rather, the injury produced by any given protocol depended on the interaction between the thermal source, the geometry and conditioning of the device, the characteristics of the tissue interface, the anatomical substrate, and the timing and method used for post-burn verification. This pattern was consistent across modalities, although the dominant sources of variability differed between contact, scald, steam, and radiant systems. The relative positioning of the principal induction modalities in relation to operator constraint and reported depth-control architecture is summarized in Figure 1.

### 3.2. Contact-Burn Models

Contact burns constituted the most extensively represented category of experimental burn induction. These models generally used a heated solid applicator, usually metallic, brought into direct contact with the skin for a defined period. Within this group, the literature could be organized into three related subtypes: conventional heated-metal contact systems, instrumented or force-constrained systems, and models using a controlled heating environment before application.

#### 3.2.1. Conventional Heated-Metal Contact Systems

Conventional contact-burn models use a heated solid applicator applied directly to the skin for a predefined duration. Their principal advantage is control of the surface footprint through applicator geometry. Their principal limitation is that burn depth may still vary with pressure, alignment, contact stability, and thermal equilibration, even when nominal temperature and exposure time are fixed.

Using a cylindrical rod heated by boiling water, a rat model developed by Cai and colleagues exhibited a differentially measured wound area immediately after contact of 0.9957 cm^2^ with a standard deviation of 0.1845, corresponding to a coefficient of variation of 18.5% [28]. Histological depth increased as contact time lengthened, with mean depths of 1.30 mm at 5 s, 2.35 mm at 10 s, and 2.60 mm at 20 s [28]. Dispersion was not uniform across exposure durations: variability was lower at 10 s than at 5 s or 20 s. This suggests that the precision of a contact-burn protocol may vary across its exposure range rather than remaining constant across all temperature–time combinations.

Earlier rat standardization studies also supported the feasibility of generating reproducible contact burns when applicator geometry and exposure duration were constrained. Meyer and Silva described a standardized rat burn model based on controlled contact conditions, while later work by Venter et al. showed that stepwise increases in contact temperature under fixed exposure conditions could generate progressively deeper injuries with histological separation between severity groups [21,42]. Taken together, these studies suggest that conventional contact systems can provide useful experimental control, but their performance depends on more than nominal device settings. What appears standardized at the level of design may still remain vulnerable to unmeasured differences at the tissue interface.

#### 3.2.2. Instrumented and Force-Constrained Contact Systems

A second group of contact-burn models was developed specifically to reduce operator dependence by constraining force, alignment, and temperature more directly. Instrumented and force-constrained systems were developed to reduce variability introduced by manual application. These systems aim to standardize variables such as applied force, perpendicularity, contact duration, and applicator temperature, thereby reducing differences in delivered thermal dose between repeated applications.

Arda et al. designed an apparatus intended to standardize experimental burn induction and demonstrated that applied force significantly altered burn depth even when temperature was held constant [36]. As shown in this model, higher force resulted in greater depth of injury at both 60 °C and 80 °C, indicating that pressure is a major determinant of delivered injury severity rather than a minor procedural detail [36]. This has significant implications regarding reproducibility; when pressure is operator-dependent, nominally identical device settings may still produce different injury depths if applied force is not constrained or reported.

The same pattern can be seen in porcine contact systems developed for controlled partial-thickness injury. Gaines et al. described a porcine deep partial-thickness burn model in which tighter control of applicator performance and contact conditions helped reduce procedural drift at the point of energy transfer [43]. The importance of these systems lies not only in technical refinement. They also move the model closer to a true interface-controlled approach, in which reproducibility depends on controlling the variables most likely to influence heat delivery during actual tissue contact.

#### 3.2.3. Controlled Heating-Environment Contact Models

A further development in contact-burn methodology has been the use of a controlled heating environment before the applicator is brought into contact with the skin. In these systems, the applicator is held at a predefined temperature for a specified period, usually within an oven or another regulated device, so that temperature at the time of use is more stable and less affected by incomplete equilibration.

Guo et al. described a rat model of deep partial-thickness burn in which temperature, pressure, and application conditions were controlled in an effort to improve consistency [29]. Their study highlighted the importance of standardized force and thermal preparation in producing a more uniform injury and in reducing unwanted variation in burn severity over time [29]. A similar principle was reported more recently by Ibrahim and colleagues, who used a controlled-heating system and showed that a stainless-steel rod applied for 30 s produced mean injury depths of 178 ± 46.6 μm at 100 °C, 371.2 ± 41.3 μm at 150 °C, and 385.2 ± 38.0 μm at 200 °C [22]. Although the difference between the two higher-temperature groups was smaller than that between the lower and intermediate settings, the findings still support the view that burn depth can be modulated under tightly controlled exposure conditions.

A related pattern was seen in the porcine work of Papp et al. Their study showed that a repeatable contact-burn protocol could generate similar depth categories across subjects, but it also demonstrated that burn depth continued to evolve after the initial injury [37]. This is an important point, because it connects two aspects of reproducibility that are often considered separately: the consistency of the induction step itself and the consistency of the timepoint at which severity is verified. A protocol may be highly repeatable at the moment of application, yet still lead to different depth classifications if wounds are assessed at different post-burn intervals.

Overall, the contact-burn literature suggests that achieving stable wound area is generally easier than achieving stable wound depth. When contact geometry is fixed, the surface footprint can often be reproduced with acceptable consistency. Depth is less straightforward. It remains sensitive to thermal equilibration, pressure, alignment, tissue thickness, and the timing of validation.

### 3.3. Scald-Burn Models

The second major group of burn-induction methods consisted of scald models. These were especially relevant in studies designed to produce larger areas of injury or to reproduce hot-liquid exposure of greater clinical relevance. In comparison with contact systems, scald methods often provided more even exposure across a predefined skin surface. Their reproducibility, however, depended on a different set of variables. Immersion conditions, template seating, water stability, and narrow timing thresholds all emerged as important sources of variation.

#### 3.3.1. Murine Template-Based Scald Systems

In mice, scald protocols were often designed around a template or exposure window that isolated a defined skin area while protecting the surrounding tissue. This approach helped improve consistency in wound area and limited unwanted extension of the burn beyond the intended field. Medina et al. developed a standardized deep partial-thickness scald model in C57BL/6 mice and showed that even small differences in exposure time at a fixed temperature could shift the injury from one depth category to another [30]. In that study, exposure to 54 °C water for 18 s produced superficial partial-thickness injury, 20 s produced predominantly deep partial-thickness injury, and 22 s resulted in full-thickness injury according to the pathology scoring system that was used [30].

This narrow transition range is methodologically important because it shows both the strength and the limitation of scald models. On one hand, these models allow a high degree of tuning. On the other, that tuning operates within a relatively narrow practical window. Under those conditions, reproducibility depends not only on the set temperature, but also on precise timing, proper seating and sealing of the template, and uniform contact between the exposed skin and the heated liquid. In practice, this means that scald protocols may appear straightforward while still requiring very tight procedural control.

#### 3.3.2. Porcine Scald Models and Circulating-Water Systems

Porcine scald systems offered a different kind of methodological advantage. The larger skin surface available in these models allowed multiple standardized burns to be created on the same animal, which improved within-study comparison and made repeated assessment over time more feasible. At the same time, the porcine literature also illustrated how difficult it can be to assume equivalence across protocols. In their systematic review of porcine burn conditions, Andrews and Cuttle highlighted the wide range of temperature–time combinations that had been used to generate nominally similar severity categories across studies [23]. This finding suggests that comparable burn labels cannot be inferred from temperature and duration alone.

A more controlled porcine comparison was reported by Singer et al., who examined scald and contact burns under matched nominal exposure conditions and found that the mode of heat transfer influenced later scar depth [38]. Despite apparent similarity in the thermal settings used, scald injuries healed differently from contact burns, and in that model they produced deeper scar outcomes [38]. This observation is important because it supports one of the main conclusions of the present review: burn-induction modalities should not be treated as biologically equivalent simply because they share the same nominal temperature and exposure time. Meaningful comparison requires calibration that is specific to the mechanism of injury, not just to the nominal exposure setting.

The temporal evolution of injury in heated-liquid systems also aligned with classical work on burn progression. Regas and Ehrlich showed in a rat model that vascular responses and tissue zones after thermal injury evolve over time, helping to define the logic of burn progression rather than a fixed injury state at time zero [44]. Although not a modern engineering study, that work remains methodologically relevant because it underpins the need to standardize the timepoint at which burn depth is verified.

#### 3.3.3. Rigid-Window and 3D-Printed Scald Platforms

Recent work has moved scald induction toward more constrained engineering solutions. described the Rat Printed Induction Device–3D (RAPID-3D), a custom, non-commercial 3D-printed scald-burn platform designed using SolidWorks 2024 (Dassault Systèmes, Vélizy-Villacoublay, France) and 3D-printed with support from Symme3D (Timișoara, Romania), rather than being sourced as a commercially available device [31]. In that model, exposure to boiling water for 8 s produced a mean burn area of 198.00 mm^2^ with a standard deviation of 3.54 mm^2^, corresponding to very low inter-animal variability in wound area [31]. Histological assessment demonstrated injury depths in the range of 600–900 μm, consistent with deep dermal damage [31].

The value of this study lies in how it changed the pattern of variability rather than in eliminating variability altogether. By fixing the exposure geometry and immobilizing the target area, the device reduced operator-related variation in wound area to a substantial degree. As a result, the remaining variability was more likely to reflect factors such as thermal uniformity, timing precision, and biological response, rather than inconsistency in manual contact or positioning. This pattern appears repeatedly in the literature. Better device design does not remove variability completely, but it can shift it away from uncontrolled procedural error and toward sources that are more biologically meaningful and easier to interpret.

### 3.4. Steam and Radiant Models

Steam-based and radiant models were less commonly used than contact or scald systems, but they offered an important alternative by reducing, or in some cases eliminating, direct contact pressure at the tissue surface. This changed the structure of the exposure problem. The models were constructed such that the driving sources of variability came largely from source-to-skin geometry, local environmental conditions, and output consistency, with slightly less driving influence being exerted by contact stability and/or force.

Porumb et al. described a rat steam-burn model in which exposure duration was tightly controlled [32]. Under those conditions, a 1-s steam exposure produced superficial second-degree injury, 3 s produced deep second-degree injury, and 7 s resulted in third-degree injury on histological evaluation [32]. This graded response showed that steam systems can support intentional modulation of burn depth when output is stable and exposure time is carefully controlled. At the same time, the reproducibility of such systems still depends heavily on practical details, particularly nozzle position, source-to-skin distance, and the consistency of steam delivery during each application.

Radiant systems provided a different non-contact approach. Güzey described an infrared heater model in rats in which injury severity depended on both exposure duration and the fixed distance between the heat source and the target tissue [33]. In that model, shorter exposures produced partial-thickness injury, whereas longer exposures resulted in full-thickness injury, with corresponding histological differences between groups [33]. The study also showed, however, that even small changes in distance and local ambient conditions could alter the thermal environment during exposure [33]. This is methodologically important. Although radiant systems remove the confounding effect of direct contact pressure, they remain highly sensitive to geometry, shielding, airflow, and local heat accumulation.

Overall, steam and radiant systems reduce variability related to direct contact pressure, but they do not eliminate procedural uncertainty. Instead, the dominant sources of variability shift toward source output, source-to-skin distance, angle, shielding, airflow, and environmental control. These systems therefore require calibration and reporting of spatial and environmental exposure conditions.

### 3.5. Burn-Depth Validation Methods

Across modalities, histopathology remained the predominant method for validating burn depth. It was the most frequently used reference standard in studies aiming to distinguish superficial partial-thickness, deep partial-thickness, and full-thickness injury [22,28,29,30,31,32,33,34,35,36,37,38]. Histology was often combined with semiquantitative grading, and in some studies with direct morphometric measurement of injury depth in micrometers or as a proportion of dermal thickness [22,28,31].

The strength of histological validation was its ability to anchor severity to tissue structure rather than to nominal exposure conditions. Its limitation was that the interpretation of burn depth depended strongly on the timepoint selected for assessment. Papp et al. showed that burn depth continued to progress during the first 48 h after contact injury [37]. Similar temporal behavior was observed in porcine scald work summarized by Andrews and Cuttle [45]. These findings support the view that burn depth should not be treated as an intrinsic property fixed at the moment of exposure, but as a time-dependent outcome that requires standardized verification. The principal validation approaches identified across modalities are summarized in Table 2.

Beyond histology, several studies incorporated quantitative imaging or perfusion-sensitive methods to improve severity assessment. Mazhar et al. used spatial frequency domain imaging in a porcine model and showed that non-contact optical assessment could discriminate burn extent and depth more objectively than unaided observation alone [30]. Gibson et al. further demonstrated that even experienced observers showed limited agreement in exact depth classification of porcine burn images, with poor reliability for unaided visual estimation despite specialist expertise [34]. These results emphasize that observer experience does not eliminate interpretive variability.

This point was reinforced by Ponticorvo et al., who compared clinical observation with spatial frequency domain imaging, laser speckle imaging, and thermal imaging in a preclinical model and showed that optical methods improved severity discrimination relative to clinical assessment alone [41]. Longitudinal work by Ponticorvo et al. also showed that SFDI and laser speckle imaging could track time-dependent changes in perfusion and healing in a rat burn model, thereby extending validation beyond single timepoint histology to functional assessment over time [40].

Taken together, the validation literature supports a layered approach. Histology remains the principal structural reference standard, but quantitative morphometry and optical or perfusion-based tools can reduce interpretive subjectivity and improve longitudinal characterization of wound progression.

### 3.6. Evidence Related to Operator Dependence

Evidence of operator dependence was present across all major burn-induction modalities, although the specific procedural steps differed between systems. In contact models, the dominant operator-sensitive variables were force, alignment, contact stability, and the interval between heating and tissue application. Arda et al. showed directly that burn depth varied with applied force under otherwise fixed conditions [36]. Cai et al. demonstrated that even in a simple rod-based rat model, dispersion in wound dimensions and histological depth persisted across repeated applications [28]. In Papp et al., the need to interpret injury at standardized post-burn intervals added a second layer of variability, because inconsistent timing of assessment could alter the observed severity class even if the induction step itself had been tightly controlled [37]. The main operator-dependent steps and the mitigation strategies reported across modalities are summarized in Table 3.

It is important to distinguish directly measured operator-related effects from operator-sensitive variables inferred from procedural design. Direct evidence was available when a study experimentally tested a procedural variable, such as applied force, or when observer agreement in severity classification was measured. Arda et al. provided direct evidence that applied force altered burn depth under otherwise fixed conditions [36], while Gibson et al. provided direct evidence that visual classification of burn depth is subject to interobserver variability [34]. In contrast, variables such as applicator tilt, template seating, nozzle position, source-to-skin distance, and shielding were frequently identified as operator-sensitive because they plausibly affect heat transfer, but they were not consistently tested as independent operator-level variables. Therefore, the term “operator-sensitive” is used in this review to include both directly measured operator effects and plausible operator-mediated procedural sources of variability.

In scald models, operator dependence was expressed differently. The most important variables were template seating, seal integrity, timing accuracy, and the uniformity of skin exposure to the heated liquid. Medina et al. showed that a difference of only two seconds at 54 °C could shift the injury from deep partial-thickness to full-thickness [30]. In such a system, minor deviations in handling are not trivial. They can alter the biological category of the wound. In more engineered scald systems, such as the RAPID-3D platform, rigid geometry reduced the degrees of freedom available to the operator and markedly improved areal consistency [31]. The remaining variability then arose more from thermal and biological factors than from manual inconsistency in setup.

In steam and radiant systems, the operator-related sources of variability took a different form. In steam models, reproducibility depended largely on consistent nozzle positioning, stable source-to-skin geometry, and reliable output over very short exposure intervals [32]. In infrared and other radiant systems, the key variables were distance, angle, shielding, and local ambient conditions [33]. These approaches reduce some of the errors associated with direct contact, but they remain highly sensitive to spatial calibration.

Anatomical site also emerged as an important source of variation. Burn induction is not applied to a uniform substrate, and differences in skin thickness, dermal architecture, and local tissue mechanics can influence the severity of injury produced by the same nominal protocol. This is supported both by theoretical work on thermal injury [46,47] and by more recent porcine studies showing that wound-healing trajectories can differ across anatomical locations even in otherwise standardized models [24,39]. For that reason, anatomical site should be treated as a methodological variable in its own right rather than as a neutral background characteristic.

A further level of operator dependence lies in how the outcome is interpreted. Gibson et al. reported poor interrater reliability for exact visual classification of porcine burn depth [34]. This is an important finding because it shows that operator influence does not end at the point of injury creation. It extends into endpoint definition unless objective validation methods are used. In practical terms, reproducibility in experimental burn research depends on both parts of the model: controlled delivery of thermal injury and controlled verification of the injury that has been produced.

Taken together, the evidence points to a consistent pattern. Burn models appear most reproducible when device geometry, force, timing, and thermal conditioning are explicitly constrained, when anatomical site is standardized, and when severity is assessed at predefined post-burn intervals using objective or semiquantitative methods. When these elements remain implicit or are left to operator discretion, reproducibility declines and comparisons across studies become more difficult to interpret.

**Figure 2:**
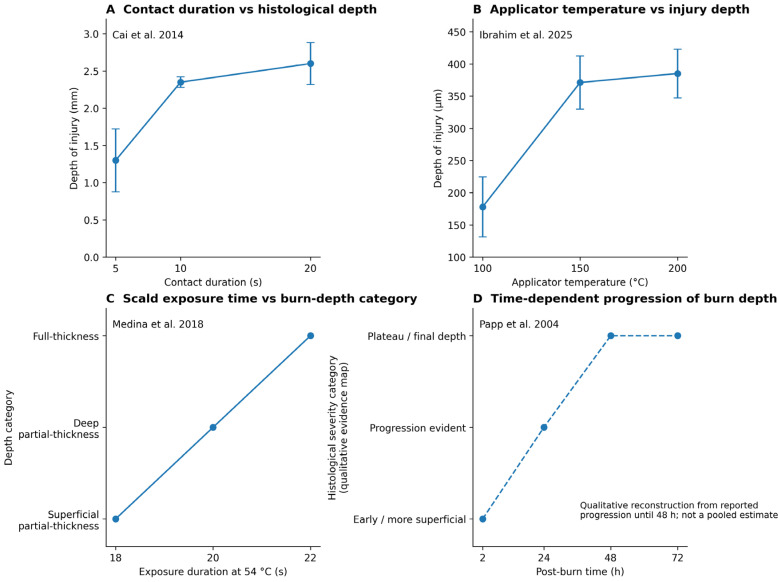
Representative quantitative and qualitative exposure–outcome relationships in experimental burn-induction studies. (**A**) Histological burn depth as a function of contact duration in a rat contact-burn model [28]. (**B**) Injury depth as a function of applicator temperature in a controlled-heating contact model [22]. (**C**) Burn-depth category transition with increasing scald exposure duration at fixed water temperature [30]. (**D**) Qualitative evidence map summarizing the reported time-dependent progression of histological burn depth after injury [37]. (**A**–**C**) summarize representative extractable quantitative or ordinal exposure–outcome data from selected model-development studies. (**D**) is schematic and should not be interpreted as a pooled quantitative estimate. The figure is descriptive and not intended as a meta-analysis, because the underlying studies differ in species, induction method, anatomical site, exposure architecture, and validation timing.

A descriptive exposure–outcome evidence map was produced to improve the visual presentation of representative relationships extracted from selected model-development studies (Figure 2). These plots were not intended for pooled statistical inference, because the underlying studies differed substantially in species, anatomical site, induction method, device architecture, exposure conditions, and validation method. Instead, the purpose of the figure was to illustrate the direction and relative magnitude of selected exposure–outcome relationships reported in the literature. Across the plotted studies, burn depth varied with contact duration, applicator temperature, exposure duration in scald models, and post-burn assessment timing. The time-dependent panel further illustrates why burn-depth classification should be linked to a predefined post-burn validation timepoint.

### 3.7. Structured Summary of Reported Reproducibility Metrics

Although pooled quantitative synthesis was not appropriate, reported dispersion and reliability metrics were extracted and summarized descriptively by modality and level of procedural constraint (Table 4). The available metrics were heterogeneous and were not reported consistently across studies. Standard deviations and coefficients of variation were most commonly available for continuous outcomes such as wound area or histological depth. Reliability statistics such as kappa or intraclass correlation coefficients were reported less frequently and were mainly relevant to observer agreement or classification of burn depth rather than to the induction procedure itself.

The extracted data suggest that area reproducibility was more often quantified than depth reproducibility. Contact models provided some of the clearest quantitative examples of depth modulation with exposure duration or applicator temperature, but they also demonstrated sensitivity to force and contact conditions. Scald models using rigid exposure windows or template-based devices reported low variability in wound area when geometry was tightly constrained. Steam and radiant models demonstrated depth tunability with exposure duration or distance, but quantitative reproducibility metrics were less commonly reported. These findings support descriptive comparison across modalities, but they do not justify pooled ranking of modalities by reproducibility.

The reviewed literature suggests that different reproducibility metrics are appropriate for different outcome types. For continuous outcomes, such as wound area, histological injury depth, or morphometric depth, SD and CV were the most commonly reported and were useful for describing within-model dispersion. CV is particularly useful when comparing relative variability across outcomes measured on different scales, but only when outcome definitions and assessment timepoints are comparable. For categorical burn-depth classification, kappa statistics are more appropriate because they quantify agreement beyond chance. For continuous ratings or repeated quantitative measurements across operators or observers, intraclass correlation coefficients are preferable. In the reviewed literature, SD and CV were more commonly available than ICC or kappa. Therefore, the evidence does not allow one metric to be identified as universally superior; rather, metric choice should match the outcome type and the source of variability being assessed.

## 4. Discussion

This review indicates that the central challenge in experimental burn induction is not the lack of available models, but the lack of a sufficiently explicit framework for understanding how injury severity is generated, constrained, and verified. The literature reviewed here makes it clear that thermal injury in laboratory animals cannot be described adequately by temperature and exposure time alone. Across contact, scald, steam, and radiant systems, the injury produced depended on a broader set of interacting variables, including heat-source behavior, interface conditions, tissue characteristics, and the timing of post-burn assessment. The conclusions of this scoping review should be interpreted at two levels. The first level consists of findings directly supported by the mapped literature: experimental burn-induction studies are heterogeneous; exposure duration, temperature, force, geometry, anatomical site, and validation timing influence reported injury severity; wound area is often more readily constrained than burn depth; histology remains the dominant structural validation method; and operator-sensitive steps are incompletely reported across modalities.

The second level is the interpretive framework proposed by the authors. The description of burn induction as a measurement-system problem is not presented as a pooled quantitative conclusion, but as a conceptual synthesis derived from the recurring observation that nominal exposure parameters alone do not fully determine delivered injury severity. This framework is intended to help organize the sources of variability identified in the literature and to guide future reporting and model-development studies. It should therefore be interpreted as a proposed methodological framework rather than as a validated quantitative ranking of burn-induction systems. In that sense, methodological rigor in experimental burn induction is best understood as a measurement-system problem. The repeatability of a model depends on how consistently thermal dose is delivered under nominally identical conditions, whereas reproducibility depends on how well that same dose can be maintained when operators, sessions, devices, or local conditions vary. That interpretation is fully consistent with contemporary understanding of burn injury as a dynamic and biologically evolving process rather than a purely instantaneous thermal event [48,49]. The translational importance of this issue extends beyond burn pathophysiology alone, because severe burns frequently require excision, grafting, dermal replacement, and scar-oriented reconstructive strategies; accordingly, methodological variability at the induction stage can propagate into downstream studies of repair and regeneration.

In contact systems, these differences are often driven by factors such as applied force, perpendicularity, contact stability, heat loss from the applicator, and incomplete thermal equilibration [36,37,49]. In scald models, the corresponding sources of variation are different, but they are equally important. Template positioning, seal integrity, uniformity of immersion, and narrow timing thresholds can all influence the final injury produced [30,31,38,45]. Steam and radiant systems reduce the role of direct contact pressure, but they introduce a different type of sensitivity, particularly to nozzle position, source-to-skin distance, angle, shielding, and ambient conditions [32,33]. Despite differences in modality, all burn-induction systems share a common methodological vulnerability: variability is introduced at the point where thermal energy is transferred to tissue. Therefore, a burn model should not be defined only by the heat source or nominal exposure parameters, but by the full set of variables that determine the thermal dose actually delivered to the tissue.

One of the clearest patterns to emerge from the reviewed evidence is that wound area is generally easier to standardize than burn depth. This is not unexpected. Surface area can often be constrained mechanically through fixed applicator geometry, rigid templates, predefined windows, or immobilization platforms. Depth is more resistant to simplification. It is shaped not only by the intended exposure parameters, but also by the physical properties of heat transfer and the biological characteristics of the target tissue. Skin thickness, adnexal structure, baseline perfusion, hydration state, and local mechanics all influence how much energy is retained and how far injury extends into the dermis and subcutis [46,47]. That distinction is methodologically important because some reports imply standardization on the basis of a stable wound footprint or a tightly specified exposure condition, while providing limited quantitative evidence that depth itself was reproducibly generated. The present synthesis suggests that this is an important point of caution. In experimental burn research, areal consistency should not be taken as evidence of severity consistency. This distinction is especially relevant for reconstructive studies, since models with stable wound area but poorly controlled depth may still yield non-comparable outcomes in graft incorporation, dermal matrix integration, contraction, and scar formation.

An additional important conclusion from this study is that there is a correlation between the depth of the burns and time as indicated in the graphic in Figure 2. This conclusion has great clinical relevance and is often underestimated when researchers perform their methodologic assessments. Burn conversion is widely accepted in the medical literature and defines a continually deepening or converting burn wound while the tissues were either viable or nearly viable at the time of the original burn injury. Ischemia, inflammatory process, oxidative stress, and failure of the microvasculature are contributory mechanisms of burn wound conversion. While there are several studies employing an animal model to analyze burn wound conversion, the data retrieved in those studies supports the premise of burn conversion supports the conviction. Burn depth can be determined based on the time of the assessment and when the evaluation is made. Papp et al. showed that histologically defined depth will continue to progress over time (i.e., a severely burned person will have some of their previously unexplained burns convert to being minimally burned 24 h after the injury). Therefore, the depth of injury classification is a function of the time of injury and the time of evaluation [37]. In scald systems, both the porcine literature and the mechanistic work underlying zone-of-stasis concepts support the view that injury severity is not fixed at the moment of exposure [23,44,45]. This has direct consequences for model interpretation. A statement that a protocol produces a “deep partial-thickness” or “full-thickness” burn is incomplete unless the post-burn timepoint used for that classification is stated explicitly. Without that information, inter-study comparisons are unstable because studies may be describing different temporal stages of the same injury process rather than genuinely different induction methods.

This naturally brings the discussion to validation. The evidence reviewed here confirms that histopathology remains the reference standard for structural assessment of burn depth. It provides the most direct way to document injury to the epidermis, dermis, adnexal structures, and deeper tissues, and it remains fundamental to distinguishing partial-thickness from full-thickness models [22,28,29,30,31,32,33,34,35,36,37,38]. At the same time, histology does not eliminate uncertainty. It shifts part of that uncertainty to other stages of the process, including biopsy timing, section plane, sampling location within the wound, and the criteria used for interpretation. For that reason, the most methodologically persuasive studies in this review were generally those that did not rely on histology alone, but combined structural validation with quantitative measurement or objective imaging. Morphometric assessment of depth, planimetric analysis of wound area, and non-contact optical or perfusion-based methods all added complementary information that made the endpoint easier to interpret and less dependent on subjective judgment [31,35,40,50]. This is also in line with the broader burn-depth literature, where objective perfusion-based techniques, particularly laser Doppler imaging, have shown better diagnostic performance than visual examination alone.

The present review also draws attention to an issue that is widely recognized in practice but less consistently reported in the literature: operator dependence remains relevant even in systems that appear technically sophisticated. In manual contact-burn models, this is relatively easy to appreciate, since variation is often introduced through force, timing, angle, or hand stability [28,36]. More engineered systems do not remove operator dependence altogether. Rather, they shift it to other stages of the procedure. In constrained scald models, for example, variability may arise through template seating, setup fidelity, or exposure handling [30,31]. In steam systems, it may depend on nozzle position or the precision with which exposure is initiated and terminated [32]. In radiant systems, geometric calibration and environmental control become particularly important [33]. Operator dependence also extends beyond the induction step itself. It remains relevant during endpoint assessment. Gibson et al. showed that exact visual classification of burn depth in porcine images had limited reliability even among experienced observers [34]. This finding demonstrates that reproducibility depends not only on how the injury is induced, but also on how the resulting injury is classified. Automation should therefore not be equated with reproducibility. Automated or constrained systems may reduce selected manual degrees of freedom, but reproducibility still depends on identifying, measuring, and reporting the residual sources of variability that remain after procedural constraint has been introduced. This distinction should be considered when interpreting reproducibility claims in model-development studies.

The issue of anatomical site deserves equal emphasis. In many experimental reports, site is recorded descriptively but not treated analytically. The evidence synthesized here suggests that this is insufficient. Anatomical location affects the baseline substrate on which thermal injury is imposed. Differences in dermal thickness, adnexal density, local perfusion, and regional mechanics can alter both the initial injury and its subsequent healing trajectory [39,46,47]. Recent porcine work has reinforced that point by showing location-dependent differences in wound healing behavior, even under otherwise standardized conditions [51]. This means that site selection is not simply a reporting detail. It is part of the exposure system. When multiple wounds are created on the same animal, anatomical distribution should therefore be prospectively balanced across treatment groups, and site-specific effects should be considered in the interpretation of variability.

### 4.1. Biological Versus Mechanical Sources of Variability

The reproducibility of experimental burn induction depends on both mechanical exposure control and biological substrate variability. Mechanical variability arises from the induction system itself, including applicator geometry, temperature stability, contact pressure, template sealing, source-to-skin distance, exposure duration, and environmental conditions. These variables determine the delivered thermal dose and are, at least in principle, controllable through device design, calibration, and procedural standardization.

Biological variability arises from the tissue substrate receiving the thermal dose. Species, strain or breed, sex, age, body weight, skin thickness, hair cycle, adnexal density, dermal architecture, hydration, perfusion, and anatomical site may all influence heat transfer, injury progression, and wound healing. The reviewed literature more consistently reported species than strain-specific or breed-specific determinants of reproducibility. Rodent studies frequently used defined strains, such as C57BL/6 mice, but few studies directly compared strain effects on burn-depth reproducibility under identical exposure conditions. Similarly, porcine models were often selected for translational anatomical relevance, but breed, age, body weight, and anatomical location may still modify tissue response and healing behavior.

Accordingly, biological substrate variables should be treated as part of the burn-induction system rather than as neutral background descriptors. However, the evidence base was insufficient to rank specific strains or breeds according to reproducibility of thermal dose response. Future model-development studies should report strain or breed, sex, age, body weight, and anatomical site in sufficient detail and, where possible, examine whether these biological substrate variables modify depth, area stability, and healing trajectory under standardized exposure conditions.

**Figure 3:**
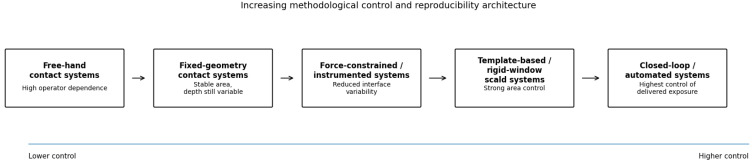
Conceptual hierarchy of methodological control in animal burn-induction systems. The figure summarizes increasing levels of procedural constraint, from poorly constrained manual systems to closed-loop or automated exposure platforms. It is not intended as a quantitative ranking of modalities by reproducibility, because the available studies differ in species, anatomical site, device design, exposure parameters, and validation methods.

The hierarchy shown in Figure 3 should be interpreted as a conceptual hierarchy of procedural constraint rather than as a quantitative ranking of modalities by proven reproducibility. The available data do not support a pooled comparison across modalities. Instead, the hierarchy summarizes the extent to which key sources of procedural variability are constrained by design, such as force control, fixed exposure geometry, template use, automation, and closed-loop temperature or exposure control.

This is also the rationale behind Table 5, which proposes a minimum reporting set for experimental burn-induction studies in animals. Its purpose is not to add unnecessary administrative detail, but to identify clearly the variables that repeatedly appeared in this review as key determinants of repeatability and reproducibility. Across modalities, the same domains recurred: device geometry, actual or interface temperature, duration of exposure, force or pressure when relevant, anatomical site, thermal conditioning, validation method, validation timing, and quantitative reporting of dispersion or reliability. These variables should be considered the minimum needed for the reader to judge whether a reported burn protocol is truly standardized or only nominally so. This proposal is also aligned with ARRIVE 2.0, which emphasizes transparent reporting of methodological details that affect reproducibility, internal validity, and interpretation of animal studies [17]. The following reporting set is proposed as a pragmatic synthesis of recurrent reporting gaps and methodological determinants identified during charting. It should not be interpreted as a validated reporting instrument or consensus guideline. Rather, it is intended as a provisional framework to improve transparency and comparability in future animal burn-induction studies. Formal validation would require a separate consensus process, such as Delphi methodology, and testing across independent model-development studies.

From a methodological standpoint, several recommendations follow directly from the reviewed literature. First, experimental burn studies should report not only the intended exposure parameters, but also the parameters most likely to alter effective dose at the tissue surface. In contact systems, these include applicator material, geometry, force or applied mass, equilibration method, and transfer interval between heating and application [21,28,36,37,43]. In scald systems, template characteristics, immersion mechanics, exposure window integrity, and water stability are equally important. For steam and radiant models, source-to-skin geometry, angle, and environmental control should be treated as central components of the induction protocol rather than as secondary setup details [32,33]. Anatomical site should likewise be predefined and justified. Severity assessment should be performed at clearly stated post-burn intervals that are biologically meaningful, and variability should be described quantitatively whenever possible, using measures such as standard deviation, coefficient of variation, kappa, or intraclass correlation coefficients rather than representative images alone. In studies where finer distinctions in burn severity are important, structural histology should be supplemented by objective or semiquantitative validation methods.

These recommendations matter for more than internal methodological elegance. They have direct implications for translational relevance. Burn injury in patients is clinically important because depth and area drive resuscitation, excision, grafting decisions, healing time, and scar burden. If preclinical models are to inform therapies aimed at limiting wound conversion, improving healing, or reducing scarring, they must generate injuries whose severity is both intentional and interpretable. A model that is easy to perform but poorly characterized may still be useful for exploratory biology. It is much less useful for cross-study comparison or translational inference. The reviewed evidence therefore suggests that the next major advance in this field is unlikely to come from inventing entirely new burn modalities. It is more likely to come from better characterization, better validation, and better reporting of the models that already exist.

### 4.2. Relevance to Soft Tissue Reconstruction and Repair

The present review primarily examined burn-induction methodology rather than the efficacy of reconstructive or regenerative interventions. Therefore, the implications for soft tissue reconstruction and repair should be interpreted as methodological and translational rather than as direct comparative evidence on grafts, dermal substitutes, or cell-based therapies. Nevertheless, the initial depth and area of a burn are important effect modifiers in studies of reconstruction and repair. Superficial partial-thickness, deep partial-thickness, and full-thickness wounds differ in residual adnexal structures, vascular supply, inflammatory burden, contraction tendency, re-epithelialization potential, and scar trajectory. These differences may influence graft take, dermal matrix incorporation, wound contraction, epithelial coverage, and scar quality. For this reason, inconsistent burn induction can propagate into downstream intervention studies: if experimental groups differ unintentionally in initial burn depth, differences in healing, graft integration, or scar outcome may reflect baseline injury heterogeneity rather than treatment effect. Accordingly, the reconstructive relevance of burn-induction standardization lies not in proving that one induction modality is superior for all repair studies, but in ensuring that the starting injury is sufficiently defined, reproducible, and validated for the intervention being tested.

This review has several limitations. The first is the marked heterogeneity of the included literature. Species, device design, induction modality, exposure conditions, definitions of burn severity, and validation timepoints varied substantially across studies. This heterogeneity limited direct comparison and precluded quantitative pooling of effect estimates. That decision was methodological rather than pragmatic. Pooling across biologically and procedurally non-equivalent systems would have risked generating summary values with limited interpretability.

A second limitation relates to reporting quality in the underlying studies. Many articles provided incomplete information on operator training, number of operators, assignment of procedural tasks, blinding of outcome assessment, or calibration of the exposure system. Operator-level metadata were frequently sparse. In several studies, interface temperature was assumed rather than directly measured, and the rationale for the selected validation timepoint was not always explicit. These omissions constrained the ability of the present review to distinguish biological variability from variability introduced by handling or instrumentation. The review also identified limited direct evidence on strain- or breed-specific effects on reproducibility. Although species and anatomical site were commonly reported, few studies directly compared different strains, breeds, or biological substrates under identical induction conditions. Therefore, conclusions regarding biological variability beyond species and anatomical site remain necessarily cautious.

A third limitation is that the review likely remains vulnerable to publication bias toward successful or stable models. Protocols that fail to produce consistent injury, or that require substantial unpublished refinement, are less likely to appear in the indexed literature. Related to this, underreporting of unstable or abandoned configurations may lead the published evidence base to overrepresent models that appear more reproducible than typical laboratory experience would suggest. Finally, because this was a scoping review focused on methodological mapping, a formal risk-of-bias assessment was not undertaken. That choice was consistent with the review objective, but it means that the literature was characterized primarily in terms of methodological content rather than graded certainty.

A further limitation is that no formal protocol was registered or published before commencement of the review. Although the review questions, eligibility criteria, and core charting domains were defined before study selection, the absence of a publicly available protocol limits external verification of all a priori methodological decisions. To mitigate this limitation, the revised manuscript explicitly distinguishes predetermined review elements from minor refinements introduced during data charting to capture modality-specific procedural variables.

The main implication of this review is that future progress in animal burn modeling will depend less on creating entirely new modalities and more on improving the metrological clarity of existing ones. The field already possesses a workable range of experimental systems. What remains inconsistent is how these systems are described, validated, and compared. A model should not be judged only by whether it can create a burn. It should be judged by whether it can create a burn of known severity, with quantified variability, on a defined substrate, at a prespecified validation interval.

That shift in emphasis could improve both internal validity and translational usefulness. It would also align experimental burn research more closely with contemporary expectations for reproducible preclinical science. In practice, the most useful next step is not a universal preference for one modality over another. It is adoption of a minimum reporting framework that captures the variables governing delivered thermal dose, anatomical context, and validation timing. Once those elements are reported consistently, cross-study comparison will become more meaningful, and genuine differences between modalities will be easier to interpret.

## 5. Conclusions

This review shows that experimental burn induction in laboratory animals is methodologically feasible across a range of contact, scald, steam, and radiant systems, but that the reproducibility of these models depends less on the nominal exposure setting alone than on how effectively the determinants of delivered thermal dose are controlled and verified. Across modalities, the most consistent pattern was that wound area can often be standardized more readily than burn depth. Depth remained more vulnerable to uncontrolled variation at the tissue interface, differences in anatomical substrate, and inconsistency in the timing and method of post-burn assessment.

A central interpretive framework arising from this review is that experimental burn induction can be usefully understood as a measurement-system problem. Seen in this way, repeatability and reproducibility are determined by the interaction of device design, operator-mediated procedural factors, tissue biology, and the strategy used to validate injury severity. This has an important practical consequence. A protocol cannot be considered fully standardized on the basis of temperature and exposure time alone. It also needs to define the variables that govern effective thermal dose at the tissue surface and to state clearly how, and under what conditions, injury severity is confirmed.

The evidence reviewed also indicates that methodological quality improves when key operator-sensitive steps are constrained. Fixed geometry, controlled force or mass, standardized alignment, regulated thermal equilibration, predefined anatomical site selection, and explicit post-burn validation timepoints all contribute to more interpretable and reproducible models. Objective validation methods, including histomorphometry and perfusion- or optics-based approaches, further strengthen model performance by reducing dependence on subjective classification alone.

Future progress in this field will depend less on the development of entirely new burn modalities and more on the adoption of clearer methodological standards for existing ones. A proposed minimum reporting set for animal burn-induction studies should include, at minimum, the induction modality, device geometry and material, target and measured interface temperature where available, exposure duration, applied force or pressure when relevant, anatomical site, peri-procedural preparation, validation method, validation timing, and quantitative measures of variability. Wider adoption of such reporting practices would improve internal validity, reduce unnecessary experimental duplication, support more efficient use of animals, and strengthen the translational value of preclinical burn research. Wider adoption of such methodological standards would strengthen not only internal validity in preclinical burn research, but also the translational utility of animal burn models for soft tissue reconstruction and repair.

## Figures and Tables

**Table 1:** Variables extracted.

Domain	Variables Charted	Rationale
Animal and model characteristics	Species, strain or breed, sex, age, body weight, anatomical site	These factors influence skin thickness, dermal structure, perfusion, and thermal response.
Pre-procedural conditions	Hair removal, skin preparation, anesthesia, analgesia, peri-procedural temperature control	These variables may alter tissue physiology and procedural consistency.
Induction modality	Contact, scald, steam, radiant, infrared, or related thermal method	Modality determines the mechanism of heat transfer and the major sources of variability.
Device characteristics	Material, heating mechanism, applicator design, exposure-window geometry, template use, immobilization method	Device design affects thermal stability, contact conductance, area control, and operator dependence.
Exposure parameters	Target or setpoint temperature, measured interface temperature when available, exposure duration, force/pressure/mass, distance, angle, intended wound area	These variables define the nominal and effective thermal dose delivered to tissue.
Validation of injury severity	Histology, morphometry, planimetry, perfusion imaging, optical imaging, macroscopic scoring	These data establish how burn depth and wound area were verified.
Validation timing	Immediate and all reported post-burn assessment timepoints	Burn depth evolves over time; timing of verification is critical for interpretation.
Reproducibility metrics	Standard deviation, coefficient of variation, interquartile range, kappa statistics, intraclass correlation coefficients, other dispersion or reliability measures	These metrics were used to identify how precision and reproducibility were quantified.
Operator-related information	Number of operators, training, standardization procedures, blinding, assignment of induction procedures	These data were necessary to assess operator-dependent variability.
Design features intended to reduce variability	Fixed load, alignment systems, templates, rigid exposure windows, automation, closed-loop temperature or exposure control	These features were charted as reported mitigation strategies for improving repeatability and reproducibility.

**Table 2:** Burn-depth validation methods across experimental burn models.

Validation Method	What It Captures	Representative Use in the Reviewed Burn-Model Literature	Main Strengths	Main Limitations
Histology	Structural tissue injury and burn-depth category	Used as the principal validation method in contact, scald, steam, and radiant models [21,28,29,30,31,32,33,37,38,42,43]	Provides structural confirmation of partial- versus full-thickness injury	Strongly influenced by biopsy timing, sampling level, and section interpretation
Quantitative morphometry	Measured injury depth in μm or as proportion of dermal thickness	Reported in controlled rodent contact and scald models [22,28,31,37]	Allows finer discrimination than ordinal depth grading	Still dependent on timing and histologic sampling strategy
Planimetry	Wound area and contraction over time	Used to quantify wound area and longitudinal closure in rat burn models [29,31]	Useful for assessing area stability and healing trajectory	Does not independently define burn depth
Perfusion-sensitive imaging	Functional microvascular disturbance and wound progression	Incorporated in porcine and rat burn studies using optical/perfusion-based assessment [35,40,41,44]	Captures physiologic changes not visible on routine histology alone	Requires specialized acquisition and standardized imaging conditions
Optical imaging (SFDI, LSI, related tools)	Burn extent, perfusion, and objective severity stratification	Used for non-contact assessment of burn depth and extent in preclinical models [35,40,41]	Reduces observer subjectivity and supports longitudinal assessment	Technique-specific calibration and interpretation are required
Macroscopic or clinical visual scoring	Gross appearance of the wound surface	Used as a supportive assessment method in several model-development studies [23,29,33,34]	Rapid and easy to implement	Interobserver agreement is limited; exact depth assignment is unreliable [34]

Table note. Histology remained the dominant reference standard across modalities, whereas optical and perfusion-based methods were used primarily as adjuncts for objective severity discrimination and longitudinal monitoring [34,35,40,41].

**Table 3:** Operator-dependent steps and mitigation strategies by modality.

Modality	Main Operator-Dependent Steps	Principal Reproducibility Risk	Reported Mitigation Strategies
Conventional contact burns	Manual timing, variable force, tilt, perpendicularity, contact instability, interval between heating and application [21,28,36,37,42]	Depth drift despite nominally fixed temperature and exposure time	Fixed applicator geometry, use of device mass rather than hand-applied pressure, standardized timing, pre-equilibration of the applicator [21,28,36]
Instrumented or force-constrained contact systems	Residual positioning variability despite better force control [36,43]	Interface inconsistency if force or alignment is not mechanically constrained	Force-standardized devices, alignment guides, monitored applicator systems [36,43]
Controlled-heating contact systems	Transfer delay, positioning at the tissue surface, biological variability at the site of application [22,29,37]	Within-session thermal drift and inconsistent effective interface temperature	Heating in a controlled environment, defined equilibration time, standardized application conditions [22,29]
Murine template-based scald systems	Template seating, seal integrity, immersion handling, narrow timing thresholds [30]	Small timing deviations can shift injury from one depth category to another	Purpose-built templates, fixed exposure windows, strict timing control [30]
Porcine scald systems	Positioning, exposure boundary control, handling during immersion and removal [38,45]	Mechanism-specific thermal transfer and post-burn progression complicate cross-study comparability	Apparatus-level control of exposure conditions and mechanism-specific calibration [38,45]
Rigid-window/3D-printed scald systems	Setup fidelity and timing remain important, although hand variability is reduced [31]	Residual variability shifts from manual inconsistency to thermal uniformity and biological response	Rigid geometry, immobilization, predefined windows, standardized exposure platform [31]
Steam systems	Nozzle positioning, source-to-skin geometry, initiation/termination timing [32]	Output and geometric inconsistency during short exposures	Computer-controlled delivery and standardized nozzle setup [32]
Radiant/infrared systems	Distance, angle, shielding, airflow, local ambient conditions [33]	Geometry-sensitive heat delivery and between-session drift	Fixed-distance rigs, environmental control, standardized shielding [33]
Validation phase across modalities	Visual depth estimation, biopsy timing, sampling location [34,35,37,40,41]	Endpoint misclassification even when induction is well standardized	Prespecified post-burn timepoints, histology, morphometry, optical or perfusion-based validation [34,35,37,41]

Table note. Operator dependence did not disappear in more engineered systems; rather, it shifted from direct contact pressure and manual handling toward setup fidelity, spatial calibration, and the standardization of validation procedures [31,32,33,34,35,36,37,40,41,43].

**Table 4:** Structured summary of reproducibility-related evidence by induction modality.

Modality	Representative Quantitative or Reliability Evidence	Main Metric Type Reported	Evidence for Operator Dependence	Interpretation
Conventional contact burns	Cai et al. reported wound area of 0.9957 ± 0.1845 cm^2^, corresponding to a coefficient of variation of 18.5%. Histological depth increased from 1.30 ± 0.424 mm at 5 s to 2.35 ± 0.071 mm at 10 s and 2.60 ± 0.283 mm at 20 s [28].	SD, CV, histological depth measurements	Operator dependence was mostly inferred from force, alignment, timing, contact stability, and the interval between heating and application. Direct support for force sensitivity was provided by force-constrained contact studies [28,36].	Contact models can generate quantifiable area and depth outcomes, but burn depth remains sensitive to tissue-interface conditions.
Instrumented or force-constrained contact systems	Arda et al. demonstrated that applied force altered burn depth under otherwise fixed temperature conditions [36].	Experimental comparison of force conditions	Applied force was directly measured as a determinant of burn depth. Residual positioning and alignment effects were mostly inferred.	Force is a measurable determinant of delivered injury and should be constrained, calibrated, or explicitly reported.
Controlled-heating contact systems	Ibrahim et al. reported injury depths of 178.0 ± 46.6 μm, 371.2 ± 41.3 μm, and 385.2 ± 38.0 μm at 100 °C, 150 °C, and 200 °C, respectively [22].	SD, morphometric depth	Operator-sensitive sources were mostly inferred from transfer interval, applicator positioning, contact stability, and biological variability at the application site.	Temperature-dependent depth modulation can be demonstrated under controlled heating conditions, although plateauing may occur at higher thermal settings.
Template-based scald systems	Medina et al. showed categorical transitions from superficial partial-thickness to deep partial-thickness and full-thickness injury with 18 s, 20 s, and 22 s exposures at 54 °C [30].	Ordinal histological depth category	Operator-sensitive variability was inferred from template seating, seal integrity, immersion handling, and timing precision.	Small timing differences may shift the wound from one burn-depth category to another, emphasizing the need for strict procedural control.
Rigid-window or 3D-printed scald systems	Roșca et al. reported burn area of 198.00 ± 3.54 mm^2^, corresponding to a coefficient of variation of approximately 1.8%, with histological injury depth in the 600–900 μm range [31].	SD, CV, histological depth range	Operator dependence was reduced but not eliminated. Remaining sources included setup fidelity, timing precision, thermal uniformity, and biological response.	Rigid exposure geometry can improve wound-area stability, but burn-depth reproducibility still requires histological or objective validation.
Steam systems	Porumb et al. reported graded histological severity with 1 s, 3 s, and 7 s steam exposures [32].	Ordinal histological severity	Operator-sensitive variability was mostly inferred from nozzle positioning, source-to-skin distance, initiation and termination timing, and consistency of steam delivery.	Steam models can support depth tunability, but quantitative reproducibility metrics are limited and spatial calibration remains important.
Radiant or infrared systems	Güzey et al. reported severity differences according to exposure duration and fixed source distance in an infrared burn model [33].	Ordinal histological severity	Operator-sensitive variability was mostly inferred from distance, angle, shielding, airflow, local ambient conditions, and source stability.	Non-contact systems avoid pressure-related variability but require careful control of geometry, environmental conditions, and heat-source output.
Validation and observer assessment	Gibson et al. reported limited agreement for exact visual classification of porcine burn depth [34].	Observer agreement and reliability statistics	Operator-related variability was directly measured at the endpoint-assessment stage rather than during induction.	Reproducibility depends not only on how the burn is generated, but also on how burn severity is classified and validated.

**Table 5:** Proposed minimum reporting set for experimental animal burn-induction studies.

Domain	Minimum Item to Report	Supporting Rationale from the Reviewed Literature
Animal characteristics	Species, strain/breed, sex, age, body weight	Thermal response and healing trajectory differ across models and tissue substrates [39,45,52,53]
Anatomical site	Exact body location of each burn	Site influences skin thickness, wound progression, and healing comparability [24,37,39,46,47]
Pre-procedural preparation	Hair removal, skin preparation, anesthesia, analgesia	These factors affect procedural consistency and tissue condition at the time of exposure [29,30,31,36]
Modality and device design	Burn modality, device material, applicator or window geometry	Mechanism of heat transfer and apparatus design shape the delivered thermal dose [21,28,31,32,33,36,38,43]
Exposure parameters	Set temperature, measured interface temperature if available, exposure duration	Nominal settings alone do not adequately define injury severity [28,36,37,45]
Force/pressure	Applied mass, force, or pressure for contact models	Pressure materially influences burn depth under fixed temperature conditions [28,36,43]
Spatial geometry	Distance, angle, and shielding for non-contact systems	Steam and radiant models are highly geometry-sensitive [32,33]
Area control	Template design, rigid windows, intended wound size	Wound-area stability depends on constrained exposure geometry [28,30,31]
Thermal conditioning	Method of heating and equilibration before application	Surface thermal equilibration affects reproducibility of delivered injury [22,29,36,37]
Validation method	Histology, morphometry, imaging, planimetry, scoring system	Severity claims require explicit verification methodology [28,29,30,31,32,33,34,35,37,47]
Validation timing	Exact post-burn timepoints	Burn depth is time-dependent and may progress after injury [23,37,44,45]
Operator information	Number of operators, training, standardization, blinding	Operator handling and interpretation contribute materially to variability [34,36,37,39]
Dispersion/reliability statistics	SD, CV, IQR, ICC, kappa, or equivalent	Precision and reproducibility cannot be judged without quantitative spread or reliability metrics [28,31,34,37]

Table note. This proposed reporting set is derived from the recurrent methodological determinants of variability identified across the reviewed evidence and is aligned with broader reporting expectations for animal research methodology [16,17].

## Data Availability

No new data were created or analyzed in this study. Data sharing is not applicable to this article.
